# Mapping the circle of Trolard: A holistic view of its role in neurosurgical practice, anatomical perspectives and clinical applications

**DOI:** 10.1016/j.bas.2024.102789

**Published:** 2024-03-23

**Authors:** Oday Atallah, Ahmed Muthana, Vivek Sanker, Omar Wawi, Samer S. Hoz

**Affiliations:** a Department of Neurosurgery, Hannover Medical School, Hannover, Germany; b College of Medicine, University of Baghdad, Baghdad, Iraq; c Department of Neurosurgery, Trivandrum Medical College, Kerala, India; d Department of Radiology, Sana Hospital Offenbach, Offenbach, Germany; e Department of Neurosurgery, University of Pittsburgh Medical Center (UPMC), 1542 Spring Park Walk, Pittsburgh, 15064, Pennsylvania, United States

**Keywords:** Circle of Trolard, Deep cerebral vein, Basal venous circle

## Abstract

**Introduction:**

An anatomical structure that resembles the circle of Willis, the circle of Trolard is generated in the basal cistern and travels around the midbrain in a roundabout manner, passing adjacent to the lateral side of the cerebral peduncle.

**Research question:**

The primary objective of this article is to provide neurosurgeons with a comprehensive understanding of Trolard's circle, emphasizing its anatomical features and clinical significance.

**Material and methods:**

A comprehensive evaluation of the available literature pertaining to the venous circle of Trolard was conducted by conducting searches in the PubMed, Web of Science, and Scopus databases. In the present overview, the terminologies “venous circle of Trolard,” “basal venous circle,” and “basal vein of Rosenthal” were employed.

**Results:**

Upon doing a comprehensive examination of the existing literature and primary sources pertaining to the venous circle of Trolard, it was discovered that an only six studies had been conducted on this particular subject matter. We made observations regarding the anatomical characteristics of the subject and engaged in a discussion regarding their prospective applications and importance within the context of neurosurgical procedures.

**Discussion and conclusion:**

The scarcity of research on these structures is attributed to the challenges associated in studying them in vivo. Through directing focus towards these structures, our aim is to stimulate further investigation into their potential involvement in a range of neurological and neurosurgical disorders.

## Highlights

• The circle of Trolard appears in the basal cistern and circles the midbrain, passing near the cerebral peduncle's lateral side.
• This paper aims to elucidate the anatomical features of this circle and its relevance in surgical procedures, specifically the clipping of aneurysms.
• There is a need for additional cadaver research in order to gain a deeper understanding of this phenomenon.

## 1 Introduction

The circle of Trolard is an anatomical formation that is similar to the circle of Willis. It originates in the basal cistern and travels around the midbrain in a roundabout way, passing adjacent to the lateral side of the cerebral peduncle (Braun et al., 1976). The venous circle of Trolard is created by the basal veins of Rosenthal (BVR), the anterior and posterior communicating veins. The venous circle allows blood to flow in a different direction and connects the right and left sides of the deep venous system at the level of the optic chiasm and cerebral peduncles. With the help of cutting-edge neuroimaging techniques like computed tomographic venography (CTV) and three-dimensional digital subtraction venography (3D-DSV), its presence can be readily identified even if its overall appearance is inconsistent (Acerbi et al., 2018; Cullen et al., 2005).

This venous anastomosis may be significant in pathological circumstances such as brain tumors and vascular abnormalities due to the significant individual variability in the brain's venous drainage. This circle may be significant if there are no basal vein segments or if the deep vein system is exposed to high-flow conditions like arteriovenous shunts (Cullen et al., 2005). Furthermore, a haemorrhage from this venous circle could happen during procedures like an endoscopic third ventriculostomy. We believe that understanding this venous loop may be beneficial for neurosurgeons and neuroradiologists who perform work close to the base of the brain.

The literature often lacks a precise, all-encompassing, or cohesive depiction of the venous circle of Trolard. To comprehensively elucidate the anatomy of this circle, we enumerate the many appearances of the Trolard circle and their implications for neurosurgical interventions.

## 2 Methods

**Fig. 1:**
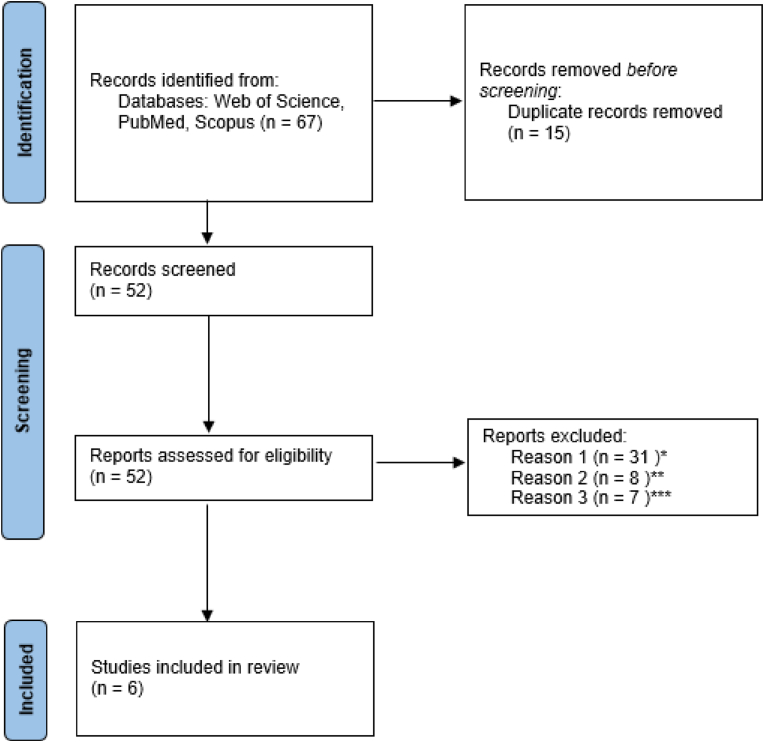
Flow diagram of the related articles * Not related articles ** Languages other than English ** Non-Human studies.

The present review undertook a thorough examination of the literature relevant to papers concerning the circle of Trolard. In order to collect relevant resources, the present investigation utilized the databases PubMed, Scopus, and Web of Science. The inclusion criterion was limited to studies that made their complete research text accessible to the public. The analysis excluded non-voll text objects and research conducted in languages other than English (Fig. 1). The present study incorporated the words “venous circle of Trolard,” “basal venous circle,” and “basal vein of Rosenthal.” The titles and abstracts of the research were examined during the initial screening phase in order to assess their suitability according to pre-established criteria. Two authors working independently (O.A., O.W.), did a comprehensive analysis by removing duplicate publications and assessing which ones met the specified criteria. During the review process, any disagreements were carefully settled by a third reviewer (V.S.) to make sure that our review was reliable and consistent.

## 3 Results

During our comprehensive analysis of accessible articles and original works, we identified six publications that specifically address the anatomical variations and neurosurgical uses of the venous circle of Trolard. The anatomical location of the circle of Trolard, its link to the circle of Willis, and the functional anatomy of the circle of Trolard were all topics that were covered in this review. The significance of these veins was also deliberated over in the context of neurosurgical procedures, including cerebral aneurysms, arteriovenous malformations, and the necessity of sacrificing them during surgical interventions. Table 1 presents a concise overview of the 6 chosen articles that offer comprehensive information regarding the venous circle of Trolard.

**Table 1:** A synopsis of the selected articles within the circle of Trolard.

Author	Title	Summary
Eskew WH (Eskew et al., 2023), 2023	The Venous Circle of Trolard: An Anatomical Study with Application to Approaches to the Basal Brain	In 42% of the specimens, Trolard's entire circle was identified. The majority of incomplete circles lacked an anterior connecting vein and were incomplete anteriorly. Above the optic chiasm, the anterior communicating veins connected to the anterior cerebral veins and proceeded posteriorly. The anterior cerebral veins were always shorter and smaller than the posterior connecting veins. Trolard's circles were generally fairly symmetrical.
Komiyama, M (Komiyama, 2017)., 2017	Functional Venous Anatomy of the Brain for Neurosurgeons	Basal Vein of Rosenthal develops following the posterior closure of the primitive tentorial sinus. Deep and superficial venous drainage are received by this vein, which is divided into three segments: diencephalic, mesencephalic, and telencephalic. Among these draining pathways, the basal vein may follow one: (i) transverse sinus (ii) superior petrosal sinus (iii) great vein of Galen (iv) straight sinus
Mukerji N (Mukerji et al., 2010), 2010	Venous anastomotic circle, multiple varices and oculomotor palsy - a rare coincidence.	Basal veins of Rosenthal, anterior and posterior connecting veins, constitutes the Venous Circle of Trolard. The anterior communicating vein and the posterior communicating vein were the two principal communicating veins in the venous rings previously described. This anastomotic circle offers a collateral venous drainage route in high-flow fistulae, and the flow direction may change. Varices may occur as a result of this irregular flow, and these infrequently result in compressive cranial nerve impairments.
Cullen S (Cullen et al., 2005), 2005	The anastomotic venous circle of the base of the brain	The right and left sides of the cerebral deep venous system are connected by an anastomotic circle of veins that is next to the arterial circle of Willis at the base of the brain. This circle is made up of the basal veins of Rosenthal and the anterior and posterior transverse anastomotic channels. In high flow conditions, such as arteriovenous shunts that access the deep venous system, or when parts of the basal vein are lacking (with or without complex DVA), the venous circle serves as a pathway for contralateral venous outflow that may become significant.
Braun JP (Braun et al., 1976), 1976	Transverse anastomoses of the veins at the base of the brain	The anterior and posterior connecting veins symbolize the venous anastomoses at the base of the brain. The quality of these transverse veins and the consistency of the anatomy are prerequisites for this anastomotic function. Contralateral venous outflow via these anterior and posterior anastomotic veins occurs under specific clinical situations due to differences in intracranial pressure.
PADGET DH (PADGET, 1956)., 1956	The cranial venous system in man in reference to development, adult configuration, and relation to the arteries.	In the description of the superficial anastomotic cerebral vein bearing his name, Trolard (1868) made reference to the tentorial sinus. He stated that the vein passed via a “veritable sinus” in the temporal fossa before emptying into the superior petrosal sinus.

## 4 Discussion

**Fig. 2:**
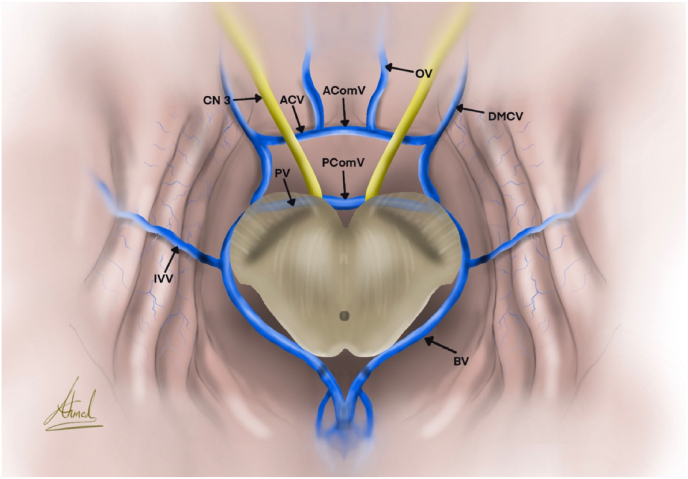
Shows the circle of Trolard. AComV: anterior communicating vein, AComV: anterior communicating vein, ACV: anterior cerebral vein, BV: basal vein, CN: cranial nerve, DMCV: deep middle cerebral vein, IVV: inferior ventricular vein, OV: olfactory vein, PComV: posterior communicating vein, PV: peduncular vein.

The anterior and posterior communicating veins, which cross the midline to connect the anterior and posterior segments of the BVR, are responsible for creating the circle of Trolard (Eskew et al., 2023) (Fig. 2). It is essential to learn how to identify the segments of BVR in order to locate the circle of Trolard, despite the fact that the cerebral veins' arrangement varies significantly.

The anterior cerebral vein of the orbital cortex and the deep middle cerebral vein of the sylvian fissure are the starting points of the BVR, which then travels along the medial border of the temporal lobe to connect to the straight sinus (Braun et al., 1976). The BVR is the main vein collecting veins from the inferior surface of the brain, and it usually flows anterior to posterior along the path of the circle of Willis. The BVR is formed by a network of veins, some of which contribute directly while others do so indirectly. These include the inferior striate veins, the deep middle cerebral vein, the inferior frontal vein, and the olfactory vein. A posterior connecting vein medially enters the middle segment of the BVR at the lateral face of the pons (PADGET, 1956). The two basal veins of Rosenthal are instantly joined by the posterior communicating vein. The medial aspect of the BVR joins with the lateral pontomesencephalic vein to form the posterior segment of the BVR. From there, the medial aspect of the BVR rotates medially to meet the internal cerebral veins and contralateral BVR at the midline. The peduncular and BVR are the components that make up the mesencephalic heart, which is shaped similarly to a heart. Through an anastomosis, this mesencephalic heart is connected to the two Rosenthal veins, which ultimately lead to the vein of Galen, where their blood is expelled. It's important to remember that the internal cerebral veins or the straight sinus are other places where the basal veins of Rosenthal may drain. The two communicating veins, the anterior cerebral veins, and the mesencephalic heart are all included in the circle of Trolard (Braun et al., 1976).

Thrombosis of the BVR can result in venous congestion and potentially cause venous infarction in the brain regions that these veins feed. The restricted drainage and accompanying hypoxia or haemorrhage can lead to different neurological impairments, depending on which specific brain areas are impacted. These deficits may include sensory impairments, hemiparesis, and changes in consciousness. Moreover, venous congestion can result in elevated pressure inside the venous system, which may potentially cause bleeding in the brain tissues (Qiu et al., 2018). It is crucial to acknowledge that the specific impairments caused by a problem with the basal veins of Rosenthal can significantly differ based on the individual's vascular architecture and the existence of other venous drainage routes that may compensate for the blockage or loss of these veins.

Approximately 50% of patients have an anterior communicating vein. Along with running parallel to it along the lamina terminalis and above the optic chiasm, the anterior communicating artery is located inferiorly and anteriorly (Komiyama, 2017). According to Komiyama, when there is a difference in size between an anterior cerebral vein and a basal vein or an anterior cerebral vein and a communicating vein, there is also typically a difference in size between the anterior communicating artery and the A1 segments (Komiyama, 2017). Alternately, the anterior communicating vein can appear as a collection of several veins of a smaller size. Afferent branches that connect to the anterior venous circle include the anterior cerebral veins, chiasmatic veins, olfactory veins, septal veins, callosal veins, and chiasmatic veins in the midline, and the inferior striate veins, insular veins, and uncal veins on the sides (Eskew et al., 2023). The significance of this vein in arteriovenous malformation (AVM) procedures is paramount, as it plays a substantial role in regulating blood flow and avoiding consequences such as vision impairment, olfactory impairment, and behavioural disorders. Surgeons need to thoroughly evaluate the structure and size of the anterior communicating vein in order to get favourable results during the operation. Furthermore, it is crucial to comprehend the many branches that are linked to this vein in order to ascertain the most effective method for managing AVM and tumors in this specific area.

The posterior communicating vein connects the middle segment of the lateral mesencephalic vein and the basal vein's posterior portion. This vein passes behind the mammillary bodies as it descends the cerebral peduncles, then through the interpeduncular space and into the longitudinal veins of the pons. The posterior perforating substance's lateral wall may sometimes be traversed by it. Seventy-five percent of people have the posterior communicating vein (Cullen et al., 2005). This vein is similar to the anterior communicating vein; however, it is longer and wider than the anterior communicating vein. The posterior communicating vein is responsible for the drainage of the premammillary, retrochiasmatic, and interpeduncular veins that are located on the floor of the diencephalon, as well as the interpeduncular vein that is located in the brainstem (Cullen et al., 2005). It may be seen in the interpeduncular fossa behind the basilar artery as a transverse connection, which is how it can be located posteriorly (Cullen et al., 2005). The posterior communicating vein is crucial in AVM and tumor procedures due to its position and role in draining vital veins in the brain. Surgeons must use caution while doing procedures in close proximity to this vein to prevent potential harm that may lead to problems such as haemorrhage or venous congestion. Comprehending the structure and importance of the posterior communicating vein is essential for achieving favourable results in neurosurgical interventions related to AVMs and tumors.

The anterior circulation is responsible for 85% of all saccular aneurysms, the most prevalent site being the anterior communicating artery (Song et al., 2020). Aneurysm rupture is the most serious complication because it leads to a high risk of death and disability (Toth and Cerejo, 2018). There is a paucity of research examining the potential consequences associated with sacrificing the veins of the circle of Trolard during clipping procedures. Nevertheless, it is of utmost importance for neurosurgeons to meticulously assess the prospective hazards and advantages associated with the sacrifice of these veins, with the aim of averting further consequences, such venous infarction or cerebral edema. Additional investigation is necessary in order to enhance comprehension of the enduring consequences and results linked to the sacrifice of the veins inside the circle of Trolard during the treatment of aneurysms.

Vascular abnormalities, such as AVMs, can have an impact on the circulus of Trolard. When an AVM is close to the circulus of Trolard, it can obstruct the veins' normal flow and cause venous hypertension (van Beijnum et al., 2011). Depending on the location and severity of the AVM, this heightened pressure may cause bleeding or neurological deficits. There have been cases of chiasmatic compression due to AVMs documented in some research studies (Sibony et al., 1982). Particularly, intra-chiasmatic AVMs can result in hemorrhaging, which can cause chiasmal apoplexy. This is because the anterior communicating segment of the circle of Trolard is located relatively close to the optic chiasm (Regli et al., 1989). As an added complication, oculomotor palsy has been linked to cases of arteriovenous fistula (AVF) connected to the venous circle of Trolard (Mukerji et al., 2010; van Beijnum et al., 2011).

The relationship between tumors, specifically meningiomas, and the circle of Trolard is of critical importance in neurosurgery. Certain types of brain tumors, including gliomas and meningiomas, can be closely associated with the circulus of Trolard. The growth of these tumors, which can obstruct normal venous drainage pathways, has also been linked to AVM (Hiyama et al., 1994; Tunthanathip and Kanjanapradit, 2020). The size, location, and invasiveness of these tumors all play a role in how they affect venous drainage and blood flow within the circle of Trolard. In cases where tumors invade the venous structures, they can cause obstruction, altering the normal flow dynamics and potentially resulting in thrombosis or venous infarction. It is important to be aware of the presence of veins in this area, particularly when there are tumors in the pineal region, pituitary gland, or medial temporal area. This knowledge is essential for recognising the possible difficulties that may occur. The circle of Trolard is a crucial route for draining veins in the brain, and any blockage or modification to this system might result in severe outcomes. Moreover, tumors in these regions might also compress adjacent tissues, resulting in elevated pressure inside the skull and worsening the disruptions in blood flow via the veins. Hence, meticulous surveillance and control of tumors located near the Trolard's circle are crucial to guaranteeing the best possible results for patients.

Neurosurgical procedures that involve the circle of Trolard have their own special considerations and difficulties. In order to protect the circle of Trolard and guarantee proper venous drainage, surgeons need to carefully plan their procedures (Cullen et al., 2005). Surgical planning is aided by preoperative evaluation, which includes advanced imaging techniques such as magnetic resonance imaging (MRI) and angiography for assessing the relationship between the tumor, AVM, or aneurysm and the circle of Trolard. Doppler ultrasound and venous indocyanine green (ICG) videoangiography are two intraoperative monitoring techniques that can provide a real-time assessment of venous patency during surgery, aiding decision-making and reducing the risk of venous injury (Acerbi et al., 2018). For the purpose of analyzing the effects of venous sacrifice, venous ICG videoangiography was repeated whenever a venous sacrifice occurred during surgery, whether it was intended or unintentional (Davidson and McComb, 2013). In all circumstances, venous ICG videoangiography allowed intraoperative real-time flow measurement of the exposed veins with exceptional image clarity and resolution. Temporary clipping tests have been found to be an easy and reversible method of checking for suspected anastomotic circulation and predicting the effect of the venous sacrifice through the identification of potential collateral circulation (Acerbi et al., 2018).

Additionally, due to its interconnectedness with other venous structures, the circle of Trolard requires a careful and individualized surgical approach. To successfully navigate during the procedure, surgeons need a thorough understanding of the anatomy and variations of the circulus of the Trolard. After surgery, it is essential to keep a close eye on the patient to detect and treat any venous drainage complications, such as venous thrombosis or venous infarction.

## 5 Conclusion

An in-depth understanding of the variations and anatomical complexities of the circle of Trolard is crucial for surgical planning and minimizing complications during procedures involving this circle. In addition, the impact of specific tumors and neurological disorders on the circle of Trolard remains an area of ongoing investigation. Understanding these mechanisms would assist in developing targeted treatment strategies and surgical interventions to preserve venous outflow and optimize patient outcomes. Additional investigation and studies using cadavers are necessary to completely understand the complex system of veins in the Trolard circle and how they might be impacted by different diseases.

## Declaration of competing interest

Oday Atallah: none.

Ahmed Muthana: none.

Omar Wawi: none.

Vivek Sanker: none.

Samer S. Hoz: none.
